# 

**Published:** 1874-05

**Authors:** 


### TABLE OF CONTENTS.

ORIGINAL COMMUNICATIONS.

The Angina of the Throat. By A. S. Payne, M.D. - - - - - - 254

Sulphate of Atropia in Ephidrosis Profusa. By C. H. Tidd, M.D. - - 285

Caffiene in Headache, (Migraine.) By T. Curtis Smith, M.D. - - - 289

What Was It? By John W. Booth, M.D. - - - - - - 294

Broncho-Vesiculitis and Bronchial Rheumatism. By L. B. Anderson, M.D. 296

CORRESPONDENCE.

Letter from A. G. Smythe, M.D., of Baldwyn, Miss. - - - - 300

Fissures in Ano Treated with Iodoform; External Treatment of Varicose Veins. 301

REMARKS, GLEANINGS AND EXTRACTS.

On Neurosal Headache—302. Ergotine Injections in Prolapsus Ani, Weeping in Children—303. Animal Vaccination; A Real Remedy for Onychia Maligna—304. Tractile Method of Reducing Strangulated Hernia; Pathognomonic Sign of Pertussis—305. By T. Curtis Smith, M. D.

Fractures of Lower Extremities Treated without Splints—305. Nux Vomica in Obstetrics; Poisoning by Rus Toxicodendron; Guarana for the cure of Sick Headache; The Betel Nut an Anthelmintic; Biliary Calculi Dissolved by Chloroform; Ablation of Mammæ Causes Sterility—306. Hypodermic Injection of Ergotine in Uterine Fibroid; Tic-douloureux cured by Ice; Treatment of Chloroform Asphyxiation—307. By A. R. Kilpatrick, M.D.

Test for Blood—308. Action of Alcohol as a Therapeutic Agent; Poisonous Analyne Dyes; Pea-nut Oil; Enemata of Bromide Potass; A Good Wash for Chilblains; Another Remedy for Hooping-cough—309. By S. M. Burnett, M.D.

EDITORIAL AND MISCELLANEOUS.

Proceedings of the Abingdon Academy of Medicine, Va. - - - 310

Fear Not while Acting Justly; Do what You Ought, Come what May - 312

It is Much Better to Reprove Openly than to be in Anger Secretly - 313

American Medical Association; Association of ex-Confederate Medical Officers; Our Original Department; Note - - - - - 314

